# Protein Surface Mimetics: Understanding How Ruthenium Tris(Bipyridines) Interact with Proteins

**DOI:** 10.1002/cbic.201600552

**Published:** 2016-12-19

**Authors:** Sarah H. Hewitt, Maria H. Filby, Ed Hayes, Lars T. Kuhn, Arnout P. Kalverda, Michael E. Webb, Andrew J. Wilson

**Affiliations:** ^1^ School of ChemistryUniversity of LeedsWoodhouse LaneLeedsLS2 9JTUK; ^2^ Astbury Centre For Structural Molecular BiologyUniversity of LeedsWoodhouse LaneLeedsLS2 9JTUK

**Keywords:** molecular recognition, protein surface recognition, protein–protein interactions, receptors, supramolecular chemistry

## Abstract

Protein surface mimetics achieve high‐affinity binding by exploiting a scaffold to project binding groups over a large area of solvent‐exposed protein surface to make multiple cooperative noncovalent interactions. Such recognition is a prerequisite for competitive/orthosteric inhibition of protein–protein interactions (PPIs). This paper describes biophysical and structural studies on ruthenium(II) tris(bipyridine) surface mimetics that recognize cytochrome (cyt) *c* and inhibit the cyt *c*/cyt *c* peroxidase (CCP) PPI. Binding is electrostatically driven, with enhanced affinity achieved through enthalpic contributions thought to arise from the ability of the surface mimetics to make a greater number of noncovalent interactions than CCP with surface‐exposed basic residues on cyt *c*. High‐field natural abundance ^1^H,^15^N HSQC NMR experiments are consistent with surface mimetics binding to cyt *c* in similar manner to CCP. This provides a framework for understanding recognition of proteins by supramolecular receptors and informing the design of ligands superior to the protein partners upon which they are inspired.

## Introduction

Protein–protein interactions (PPIs) are considered difficult to inhibit with conventional synthetic small‐molecule compounds;^1^, ^2^ they typically involve large interfaces with few discernible pockets on either protein partner—a hallmark of traditional ligandable^3^ proteins.^4^ Conventional approaches to ligand discovery, such as high‐throughput screening and fragment‐based drug discovery, have met with limited success in identifying PPI inhibitors,^5^, ^6^ but supramolecular chemical biology^7^ with a focus on understanding and controlling molecular recognition is well placed to elaborate new strategies. One such strategy is the surface mimetic approach:^8^, ^9^, ^10^ protein surface mimetics are a class of molecular structures that utilize a scaffold to project multiple binding groups over a large area of protein surface to achieve high‐affinity protein binding. Several different scaffolds have been used as protein surface mimetics, including calixarenes,^11^, ^12^, ^13^, ^14^, ^15^ porphyrins,^8^, ^16^, ^17^, ^18^ dendrimers,^19^, ^20^, ^21^, ^22^, ^23^ metal complexes,^9^, ^24^, ^25^ nanoparticles,^26^, ^27^, ^28^ and others.^29^, ^30^, ^31^, ^32^, ^33^

We and others previously introduced highly functionalized ruthenium(II) tris(bipyridine) [Ru^II^(bpy)_3_] complexes as protein surface mimetics.^35^, ^36^, ^37^, ^38^, ^39^, ^40^, ^41^ These large, multivalent, luminescent molecules have a chemically inert core, which can be peripherally functionalized with different binding groups in a stereochemically and geometrically rich manner. Hamachi and co‐workers initially designed a carboxylate‐functionalized Ru^II^(bpy)_3_ complex capable of binding to cytochrome (cyt) *c* and mediating photoreduction.^35^ Subsequently, our group and the Ohkanda group designed high‐affinity Ru^II^(bpy)_3_ complexes for binding to cyt *c* and α‐chymotrypsin.^36^, ^37^, ^38^, ^39^, ^40^, ^41^, ^42^

**Figure 1:**
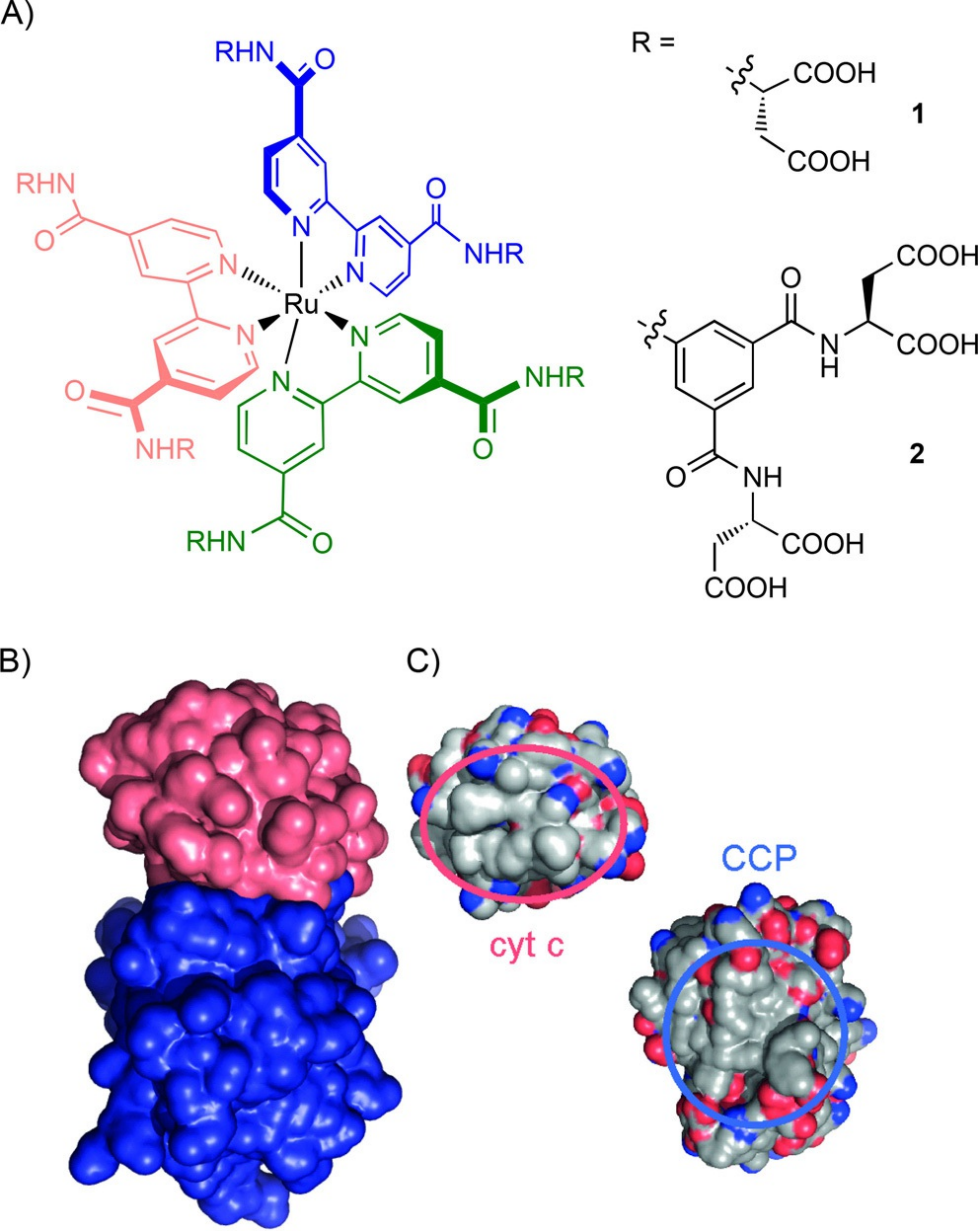
The Ru^II^(bpy)_3_ surface mimetics and their PPI counterparts cyt *c* and CCP. A) Ru^II^(bpy)_3_ complexes **1** and **2**, B) the cyt *c*/CCP interaction, with cyt *c* in pink and CCP in blue (PDB ID: 1U75),^34^ and C) the interaction faces of cyt *c* (left) and CCP (right), showing a ring (red circle) of basic amino acid residues (blue) on cyt *c* and a complementary patch (blue circle) with acidic amino acid residues (red) on CCP.

**Scheme 1:**
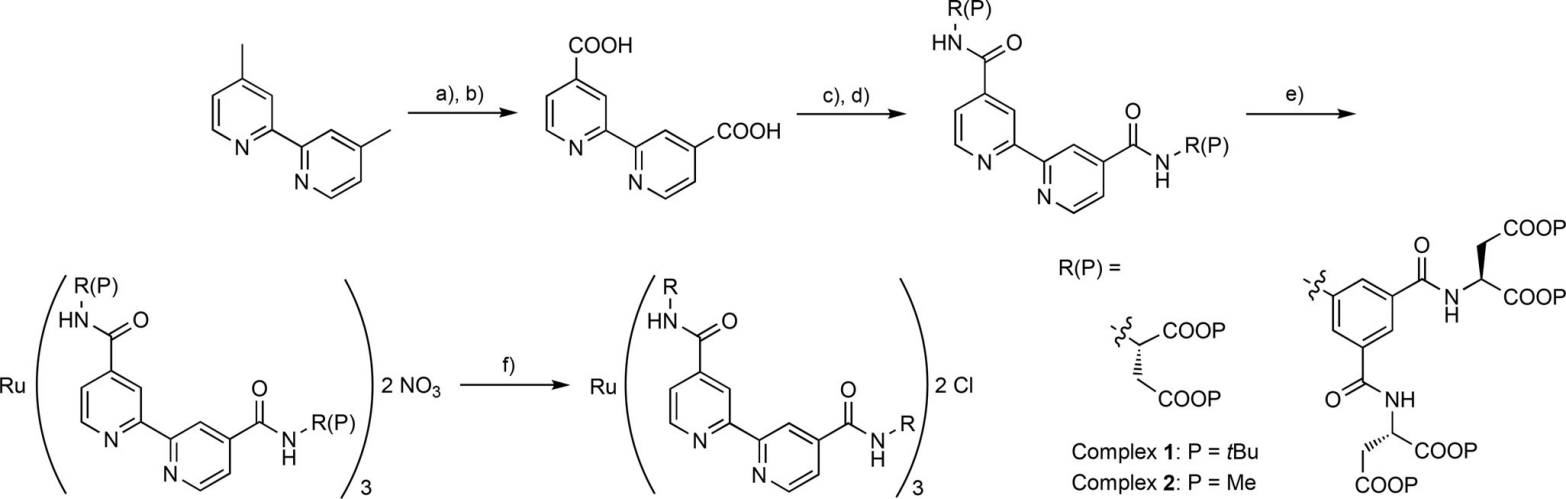
Synthesis of the Ru^II^(bpy)_3_ complexes. a) K_2_Cr_2_O_7_, H_2_SO_4_; b) HNO_3_ (84 %); c) SOCl_2_; d) CHCl_3_, DIPEA, (P)R‐NH_2_ (20–85 %); e) Ru(DMSO)_4_Cl_2_, AgNO_3_, EtOH (25–72 %); f) deprotection.

In our initial study of five different Ru^II^(bpy)_3_ complexes, two carboxylic‐acid‐functionalized complexes (Figure 1 A, complexes **1** and **2**) were shown to recognize cyt *c* with nanomolar affinity and to do with selectivity over acetylated cyt *c* and four other proteins.^36^ Complex **2** was also shown to destabilize cyt *c*.^39^ Analysis of Ru^II^(bpy)_3_ complexes with 5′‐monosubstituted bipyridine ligands (Scheme 1) showed a difference in binding affinity between *fac* and *mer* isomers (172 nm versus 25 nm for Δ isomers, respectively), but little difference between Δ and Λ isomers (25 nm versus 29 nm for *mer* isomers, respectively), thus establishing that geometrical shape affects binding.^37^ The Ohkanda group used heteroleptic complexes to show that four of the six arms of Ru^II^(bpy)_3_ complexes bearing bpy groups with two substituents interact with cyt *c*.^41^ Further studies have shown that these complexes are able to enter cells, with little cytotoxicity.^38^, ^41^

These prior strategies employed a rudimentary design that exploits charge complementarity with the cyt *c* surface;^36^, ^37^, ^43^ multiple carboxylic acids are present in order to complement surface‐exposed basic residues on cyt *c*. However, evidence of PPI inhibition,^26^, ^44^ detailed information on the nature of binding or any structural information are lacking; this is characteristic of all but a few studies on protein surface recognition by using classic supramolecular scaffolds.^18^, ^45^, ^46^ Inhibited ascorbate reduction of cyt *c* 36, 37 is consistent with binding to the cyt c peroxidase (CCP) binding site: that is, the haem‐exposed edge of cyt *c*, where there is a hydrophobic patch surrounded by a ring of basic amino acid residues.^47^ Here we show that highly functionalized Ru^II^(bpy)_3_ complexes inhibit the cyt *c*/CCP interaction and do so through electrostatically and entropically driven binding of cyt *c* in a manner that replicates the binding of cyt *c* by CCP. Higher‐affinity Ru^II^(bpy)_3_ complexes achieve additional potency through enthalpic effects. Finally, by using high‐field NMR we demonstrate that recognition occurs at the haem‐exposed edge and hence that PPI inhibition is orthosteric. Collectively, this provides a more rational framework for the design of supramolecular receptors for cyt *c* and for protein surfaces more widely.

## Results and Discussion

### Synthesis

Ru^II^(bpy)_3_ synthesis proceeded by the route shown in Scheme 1, with use of a *tert*‐butyl ester or methyl ester protecting group strategy for complex **1** or **2**, respectively. In this generic route, the ligand is first assembled by amide bond formation, via a water‐sensitive acid chloride, with subsequent complexation using Wilkinson's reagent.^48^ The protected complex formed can be purified by conventional silica flash column chromatography. Subsequent deprotection with trifluoroacetic acid (TFA) or lithium hydroxide affords complexes **1** and **2**, respectively. Deprotection of the larger complex **2** requires mild conditions and careful reaction monitoring due to the lability of the anilide bond under both basic and acidic conditions.

### Complex 2 inhibits the cyt *c*/CCP PPI

**Figure 2:**
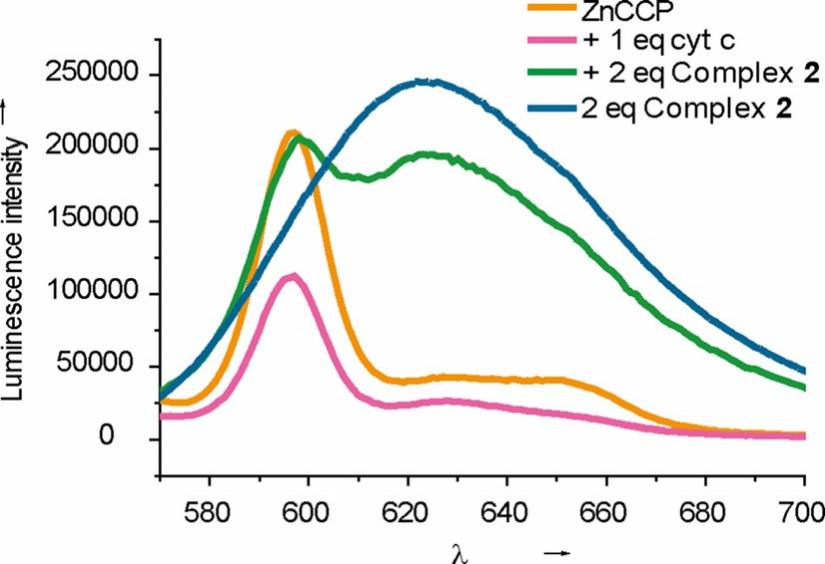
Complex **2** inhibits the cyt *c*/CCP PPI. Luminescence data (*λ*_ex_= 430 nm), 2 μm ZnCCP (orange), +2 μm cyt *c* (pink)) show loss of *λ*_max_ at 595 nm. The addition of 4 μm complex **2** (green) shows recovery of *λ*_max_ at 595 nm and reduced *λ*_max_ at 625 nm relative to 4 μm complex **2** alone (blue).

Given that the affinity of complex **2** for cyt *c* that we previously reported^36^ is greater than that of CCP for cyt *c* 49 we anticipated that **2** would be a potent inhibitor of the cyt *c*/CCP interaction. A luminescence quenching assay was implemented (Figure 2): the luminescence emission from Zn‐protoporphyrin‐substituted CCP^50^ is first quenched upon interaction with cyt *c* and then recovered upon displacement with the ruthenium complex. Signal overlap with the Ru^II^(bpy)_3_ luminescence (*λ*_max_≈625 nm) complicates interpretation; however, simultaneous loss of MLCT luminescence relative to the complex in the absence of cyt *c* is observed. A native agarose gel indicated successful PPI inhibition (see Figure S1 in the Supporting Information).

### Binding is entropically favourable and electrostatic in nature

**Figure 3:**
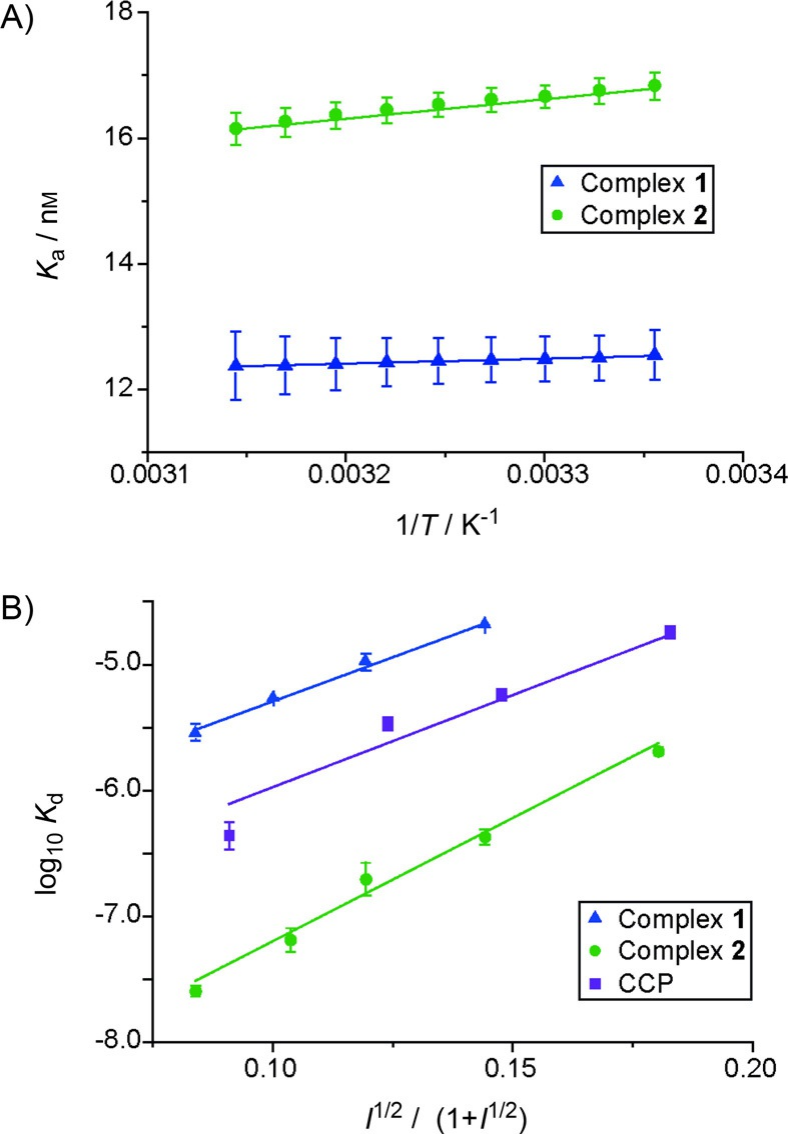
Van't Hoff and Debye–Hückel analysis on the binding interactions between cyt *c* and complexes **1** and **2**. A) Representative van't Hoff analysis (5 mm sodium phosphate, 0.2 mg mL^−1^ BSA, pH 7.5), temperature range 25 to 45 °C (errors in curve fitting for a single replicate are shown). B) Debye–Hückel analysis, with use of the Güntelberg approximation (5 mm sodium phosphate, 0.2 mg mL^−1^ BSA, pH 7.5) and variable concentrations NaCl; variation in *K*_d_ from two replicates is shown).

The binding affinities of complexes **1** and **2** towards cyt *c* were measured by means of a luminescence quenching assay,^36^ in which the luminescence of the ruthenium complexes is quenched on binding to cyt *c* through photoinduced electron transfer to its haem group. Previously, cuvette‐based fluorescence was used for binding studies;^36^, ^37^ however, optimization of the assay on a 384‐well plate was required for higher‐throughput screening of the binding under different conditions. Addition of a blocking agent—bovine serum albumin (BSA)—was found to be required to allow for agreement between the two methods. The addition of BSA accompanied a concurrent decrease in binding affinity (from *K*_d_ (10.5±0.4) nm to (42.9±3.1) nm for complex **2**, Figure S1). Determination of the *K*_d_ at different temperatures and subsequent van't Hoff analyses (Figure 3 A) provided thermodynamic parameters (Table 1) for binding [Eq. (1)], with the assumption that Δ*H* and Δ*S* are temperature independent

(1) $$\ln\;K{}_{a}=-\Delta H/RT+\Delta S/R$$

**Table 1:** Thermodynamic parameters derived from the van't Hoff analysis for the binding of complexes **1** and **2** to cyt *c* (errors derived from triplicate experiments), together with literature values for the cyt *c*/CCP interaction under similar conditions.^49^

	Complex **1**	Complex **2**	CCP^49^
Δ*H* [kJ mol^−1^ ]	−6.6±0.4	−26.3±3.0	9.4±0.8
*T*Δ*S* (25 °C) [kJ mol^−1^ ]	24.5±0.4	16.0±3.0	38.4±0.9
Δ*G* (25 °C) [kJ mol^−1^ ]	−31.0±0.4	−42.3±0.0	−27.9±1.0

These data show that, for complex **1**, binding to cyt *c* is primarily driven by entropic contributions with a small favourable enthalpic contribution, whereas for complex **2** it is both entropically and enthalpically driven. In comparison, the cyt *c*/CCP interaction is entropically driven, and enthalpically is mildly unfavourable.^49^ Thus, complex **1**, with fewer carboxylate moieties, more closely matches the thermodynamic profile of CCP in binding to cyt *c*. A plausible hypothesis for the enhanced binding of complex **2** to cyt *c* is that the additional carboxylic acids form increased numbers of salt bridges with the basic amino acids on the cyt *c* surface.

To aid further understanding of the electrostatic contribution to binding, affinities were determined at different ionic strengths (*I*). Cyt *c* binding by both complexes **1** and **2** is highly dependent upon ionic strength (Table 2), with binding affinity decreasing with increasing ionic strength, suggesting that electrostatics dominate binding. The *K*_d_ values could be fitted to the Debye–Hückel relationship [Eq. (2), Figure 3 B], in this case with use of a Güntelberg approximation [Eq. (3)], which is valid up to *I*=100 mm.

(2) logKd=logK0d0.509Z1Z2μ

(3) $$\mu \approx^{''}\surd I/\left(1+\surd I\right)$$

**Table 2:** Binding in variable ionic strengths (5 mm sodium phosphate, 0.2 mg mL^−1^ BSA, pH 7.5, variable concentrations NaCl).

Ionic strength [mm]	Complex **1** *K*_d_ [μm]	Complex **2** *K*_d_ [nm]
8.39	2.88±0.46	25.3±2.4
13.39	4.25±0.47	64.8±13.7
18.39	10.30±1.61	196.5±59.2
28.39	20.23±0.16	426.5±59.8
48.39	n.d.	2040.9±152.6

n.d.: not determined.

From this relationship the parameters K0d and *Z*_1_ *Z*_2_ can be established (Table 3), providing an estimate of the affinity at *I*=0 and the product of the interacting positive and negative charges. The data were consistent with the Güntelberg approximation for both complexes (Figure 3 B), and the calculated values of K0d show high‐affinity binding for complex **2** and weaker binding for complex **1** at zero ionic strength. The product, *Z*_1_ *Z*_2_, provides an indication of the charges involved in the interaction, with complex **2** having a larger value than complex **1** and CCP. From these data, the charge on the complex interacting with cyt *c* can be estimated. A rudimentary interpretation of this date is made possible by assuming that cyt *c* has the same charge in all cases (calculated to be ≈6 at pH 7.5);^51^ the charges on complexes **1**, **2** and CCP can thus be calculated as 4.3, 5.9 and 4.8, respectively.

**Table 3:** Parameters derived from the Güntelberg approximation of Debye–Hückel analysis for the binding of complexes **1** and **2** to cyt *c* and literature values for CCP under similar conditions.^52^

	Complex **1**	Complex **2**	CCP^52^
K0d [nm]	253±5	1.11±0.21	40.7±23.0
*Z*_1_ *Z*_2_	25.9±1.9	35.6±1.3	28.8±4.8

Complex **1** and CCP have relatively similar charges, and this suggests they make similar electrostatic interactions with cyt *c*. Complex **2** has a larger charge, indicating increased electrostatic interactions with cyt *c*. This is consistent with the van't Hoff analyses. Accounting for the crudeness of the Debye–Hückel approximation, in which small (≈3 Å), evenly dispersed charges are assumed (even when using the Güntelberg extension), the data indicate that perhaps not all carboxylate moieties are deprotonated under the assay conditions (i.e., pH 7.5) and/or that a limited number of carboxylate moieties are needed for productive protein surface recognition (even fewer than the number identified in the “deletion” study by the Ohkanda group using heteroleptic complexes).^41^

Differences in affinity between cyt *c* and complex **2** were also studied in different buffers (Table 4). Variation in affinity might discriminate between different contributions to binding because negatively charged anions must be displaced from cyt *c* and positively charged cations from complex **2**. In potassium and sodium phosphate no difference in affinity between complex **2** and cyt *c* is observed, thus indicating that interactions of the cationic buffer components with complex **2** are not significant. For binding of cyt *c* to complex **2** in phosphate or sulfonic acid buffers (MOPS and HEPES), similar affinities are also observed. This suggests that the nature of the anion and, more importantly, the hydrophobicity of the buffer are not significant in mediating molecular recognition, and reinforce the conclusions gleaned from Debye–Hückel analysis that the interaction is dominated by electrostatic contributions. For the Tris buffers (Tris and Bis‐Tris propane (btp)) a small decrease in binding affinity is observed. Although a difference in behaviour due to the chloride counter anion cannot be excluded, this might be due to the ability of btp and Tris to participate in different interactions with both cyt *c* and complex **2**; in addition to the ammonium function, the hydroxy groups on the buffer might make chelating hydrogen bonds with charged residues on either.

**Table 4:** Binding affinities for complex **2** to cyt *c* in different buffers. All buffers were at 5 mm concentration, pH 7.5.

Buffer	*K*_d_ [nm]	Buffer	*K*_d_ [nm]
sodium phosphate	42.9±3.1	HEPES	31.2±3.1
potassium phosphate	26.2±3.1	Tris	106.3±32.6
MOPS	35.2±3.1	btp	133.5±37.4

**Figure 4:**
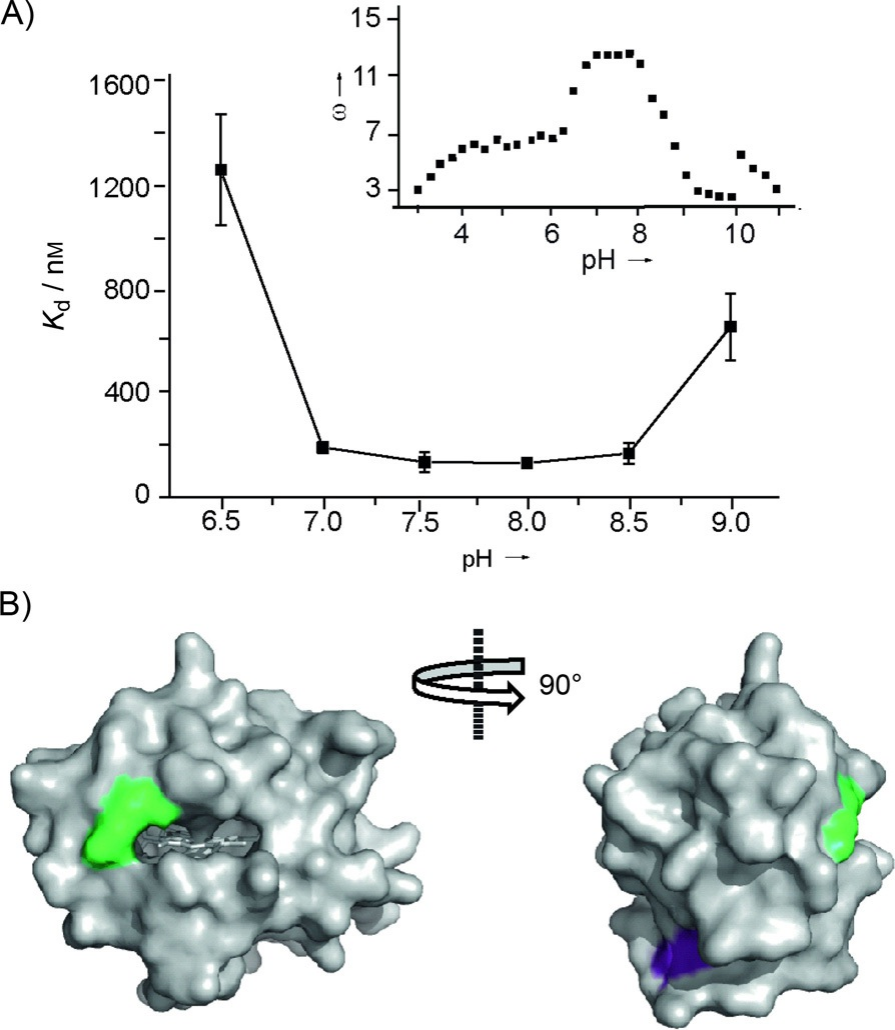
Effect of pH on the binding of complex **2** to cyt *c*. A) Binding affinity over the range pH 6.5–9.0. Inset: the electrostatic interaction factor (*ω*) of cyt *c* over a range of pH values (base limb of titration curve).^53^ B) Cyt *c* structure (PDB ID: 1U75)^54^ with residues that become protonated at pH 6.5 (His33: purple) and 9.0 (Lys79: green).

Cyt *c* is a stable protein that does not unfold over a wide range of pH values; however, its ionization state is affected by pH,^53^ so the pH of the solution was expected to affect recognition of cyt *c* by Ru^II^(bpy)_3_ complexes. To investigate the binding affinity between complex **2** and cyt *c* over a broad pH regime, btp was used, because it allows for a pH range of 6.5–9.5. The affinity follows an inverted bell‐shaped profile (Figure 4 A), which maps reasonably well onto the ionization state of cyt *c* (Figure 4 A, inset).^53^ The affinity between pH 7.0–8.5 is relatively constant with decreased binding observed at pH 6.5 and pH 9.0. Residues that become protonated/deprotonated in this pH regime are His33 and Lys79, respectively.^53^ Lys79 (green) is at the haem‐exposed edge (Figure 4 B) where binding of complex **2** is thought to occur, whereas His33 (pink) is on the distal face of cyt *c*.

A number of reasons for a decrease in binding affinity at this pH are possible: 1) Complex **2** might bind to a different or multiple sites on cyt *c*, 2) binding of complex **2** might cause subtle conformational changes that transmit to the distal face of cyt *c*, affecting the p*K*_a_ of His33, 3) protonation of His33 might cause subtle conformational changes that affect binding interactions on the haem‐exposed edge, or 4) the protonation state of complex **2** might be changed at pH 6.5. More careful analysis of the pH–*K*_d_/ionization state profiles reveals a discrepancy. The ionization state of cyt *c* drops at pH 8.0 rather than pH 8.5, at which the binding diminishes, thus suggesting that binding of complex **2** might mask Lys79 and increase its p*K*_a_. In contrast, there is no difference in the profiles for *K*_d_ and ionization state of cyt *c* in the lower pH range, thus suggesting that the p*K*_a_ of His33 is not affected by binding and that loss of affinity more likely originates from a change in ionization state on complex **2**.

### High‐field NMR reveals that the complexes 1 and 2 bind to the CCP binding site on cyt *c*

Although the pH data provide some crude structural information on the cyt *c* binding site of Ru^II^(bpy)_3_ complexes, more detailed residue‐specific, atomic‐level data were sought. To identify the binding site of complex **1** and **2** on cyt *c*, a sensitivity‐enhanced natural abundance ^1^H,^15^N HSQC spectrum of cyt *c* in the presence and in the absence of complex **1** was recorded, with a 950 MHz NMR spectrometer. Sodium ascorbate (2 mm) was added to the buffer, to reduce the iron in cyt *c* from paramagnetic Fe^III^ to diamagnetic Fe^II^, thus minimizing its influence on the spectrum (i.e., paramagnetic line broadening). The binding of the complexes to cyt *c* for reduced versus oxidized cyt *c* is similar (for complex **2**, *K*_d_=(92.4±5.5) and (49.6±13.3) nm, respectively, in 5 mm phosphate, 2 mm sodium ascorbate, 0.2 mg mL^−1^ BSA).

**Figure 5:**
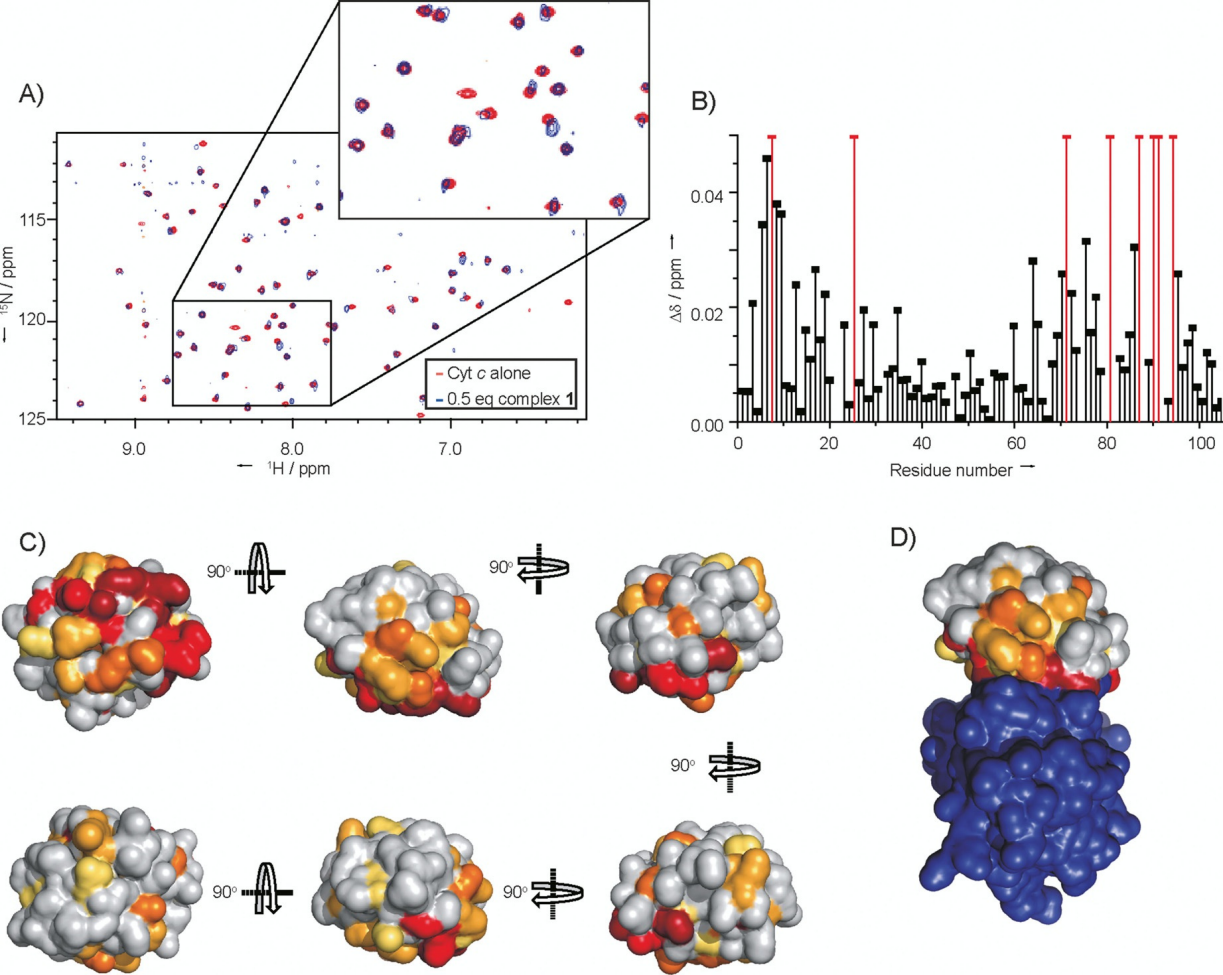
^1^H,^15^N HSQC NMR data for complex **1** binding to cyt *c*. A) Region of the overlaid HSQC spectra of cyt *c* (red) and cyt *c* with 0.5 equiv complex **1** (blue). Inset shows zoom in of part of the spectrum, showing some peaks staying the same, some having shifted and one disappearing. B) ^1^H,^15^N chemical shift differences (Δ*δ*) for the different amino acid residues with and without complex **1**. Gaps are for proline residues and unassigned amino acids; red bars show amino acids for which the signal disappears due to significant line‐broadening of NH crosspeaks on addition of complex **1**. C) Chemical shift perturbation map of cyt *c*, molecular surface of cyt *c* generated from PyMol (PDB ID: 1U75),^54^ with colouring corresponding to the extent of chemical shift changes (Δ*δ*) on addition of the complex. Amino acids with ^15^N,^1^H resonances that disappear are shown in dark red, those that exhibit large chemical shift changes (Δ*δ*>0.03) are in red, moderate changes (Δ*δ*>0.02) are in orange, small changes (Δ*δ*>0.015) are in yellow‐orange and very small chemical shift changes (Δ*δ*>0.01) are in yellow. D) Perturbation map of cyt *c* (as in (C)) in complex with CCP (purple); this view corresponds to that of the central top image in (C) (PDB ID: 1U75).^34^.

The assignment of the ^1^H,^15^N HSQC spectrum of horse heart cyt *c* has previously been achieved.^55^ After addition of complex **1**, the NMR data show that several crosspeaks have disappeared, whereas others display chemical shift changes ranging from 0.015–0.05 ppm indicating the presence of protein–ligand interactions (Figure 5 A and B). When these chemical shift changes are mapped onto the structure of cyt *c* from the cyt *c* **⋅**CCP crystal structure,^54^ the data indicate that binding occurs predominantly to one side of the haem group, with the opposite face having very few amino acids with sizeable shifts in their HSQC peaks (Figure 5 C). The binding site is in a location similar to that of carboxylic‐acid‐functionalized porphyrins.^18^ In comparison with the cyt *c*/CCP interaction (Figure 5 D), it can be seen that the amino acids for which cross‐peaks have shifted are in and around the PPI interface, thus indicating that complex **1** is an effective mimic of CCP, binding at the same face and capable of acting as an orthosteric inhibitor of the interaction.

Attempts to acquire data in the presence of complex **2** were difficult, due to the high‐affinity binding and the relatively high concentrations required for natural‐abundance NMR. At 1:1 ratios of cyt *c* and complex **2**, data could not be obtained, due to the formation of oligomers and a concomitant loss of NMR signal intensity, caused by significant line broadening. We found this unsurprising given the potential for aggregation at higher concentrations and the observation of additional binding modes in NMR studies with porphyrins.^18^ Further evidence of an additional/alternative binding mode for the larger complex **2** is given by the observation of a second binding event for complex **2** with yeast cyt *c*, but a single binding event for complex **1** (Figure S2). Even at a complex **2**/cyt *c* ratio of 1:2 multiple signals disappeared, so detailed information as to the binding site could not be gleaned; however, of the signals present, chemical shift changes were detected for regions of the protein backbone located on the same binding face as for complex **1**, and on the haem‐exposed edge.

## Conclusion

We have performed a detailed study on the cyt *c* binding properties of two synthetic Ru^II^(bpy)_3_ complexes **1** and **2**. The ruthenium complexes are potent ligands for selective protein surface recognition of cyt *c* and capable of inhibiting the cyt *c*/CCP PPI. Binding is shown to be entropically favourable and driven by complementary electrostatic interactions between the basic protein and acidic Ru^II^(bpy)_3_ complexes. This profile is consistent with accurate mimicry of the cyt *c*‐binding properties of CCP. Higher‐affinity recognition of the protein target can be achieved through the addition of further acidic motifs on the Ru^II^(bpy)_3_ complexes, allowing additional enthalpically favourable electrostatic interactions to occur. Finally, NMR experiments have established that the Ru^II^(bpy)_3_ complexes **1** and **2** bind to the solvent‐exposed cyt *c* surface, thus further underscoring the ability of the complexes to act as mimics of CCP and confirming an orthosteric mode of PPI inhibition. These studies highlight the value of detailed analyses of protein‐surface recognition by supramolecular hosts in terms of rationalizing structure–function relationships and informing subsequent designs. Moreover, the conclusions of this study point to a future need for syntheses/assembly of asymmetrically functionalized Ru^II^(bpy)_3_ complexes to maximize productive protein–ligand contacts and selectivity of protein surface recognition. This and the application of our approach to therapeutically attractive protein targets will form the basis of future studies by our group.

## Experimental Section

**Synthesis**: Synthesis was adapted from the literature.^36^ A representative synthesis of complex **1** is shown below. The synthesis of complex **2** is described in the Supporting Information.

**(2*S*,2′*R*)‐Tetra‐*tert*‐butyl 2,2′‐(((2,2′‐bipyridine)‐4,4′‐dicarbonyl)bis(azanediyl))disuccinate**: 2,2′‐Bipyridine‐4,4′‐dicarboxylic acid (100 mg, 0.400 mmol), triethylamine (1 drop) and thionyl chloride (4 mL) were heated under reflux for 16 h. The mixture was cooled to room temperature, and the thionyl chloride was removed in vacuo to yield the acid chloride as an orange‐red solid. The dry acid chloride was then redissolved in dry chloroform (20 mL) and added dropwise at 0 °C to a stirred solution of di‐*tert*‐butyl l‐aspartic acid**⋅**HCl (253 mg, 0.901 mmol) and triethylamine (0.25 mL, 1.80 mmol) in dry chloroform. The reaction mixture was warmed to room temperature and heated at reflux for 48 h. The mixture was cooled to room temperature, and the solvent was removed to yield the crude product as a brown oil. This was purified by flash column chromatography (3–6 % MeOH in CHCl_3_) to yield the product as a yellow solid (262 mg, 0.375 mmol, 91 %); ^1^H NMR (300 MHz, CDCl_3_): *δ*=1.49 (s, 18 H, H1/H2), 1.52 (s, 18 H), 2.91 (dd, *J=*17.2, 4.3 Hz, 2 H), 3.04 (m, *J=*17.2, 4.3 Hz, 2 H), 4.92 (dt, *J=*7.5, 4.3 Hz, 2 H), 7.46 (d, *J=*7.5 Hz, 2 H), 7.77 (dd, *J=*5.0, 1.65 Hz, 2 H), 8.78 (app. s, 2 H), 8.83 ppm (d, *J=*5.0 Hz, 2 H); ^13^C NMR (126 MHz, CDCl_3_): *δ*=28.0, 28.1, 37.5, 49.7, 81.9, 82.8, 118.0, 121.8, 142.3, 150.1, 156.3, 165.1, 169.5, 170.2 ppm; IR (solid state): $\tilde{\nu }$ =3346, 2975, 2928, 1723, 1650 cm^−1^; HRMS (ESI): *m*/*z* calcd for [C_36_H_51_N_4_O_10_]^+^: 699.3599; found: 699.3615.

**Tris ((2*S*,2′*R*)‐tetra‐*tert*‐butyl‐2,2′‐(((2,2′‐bipyridine)‐4,4′‐dicarbonyl)bis(azanediyl))disuccinate)ruthenium(II) dinitrate**: (2*S*,2′*R*)‐Tetra‐*tert*‐butyl‐2,2′‐(((2,2′‐bipyridine)‐4,4′‐dicarbonyl)bis(azanediyl))disuccinate (300 mg, 0.429 mmol), (dimethylsulfoxide)dichlororuthenium(II) (65 mg, 0.134 mmol), silver nitrate (46 mg, 0.268 mmol) and ethanol (20 mL) were heated under reflux for seven days, after which the reaction mixture was filtered hot and concentrated. The red solid was then loaded onto an SP Sephadex column and eluted with acetone/NaCl (0.1 m) solution (1:1), and all the red fractions were collected and concentrated. The combined red fractions were redissolved in acetone and filtered to remove sodium chloride, and this was repeated until no more white salt was visible in the concentrated sample. The complex was then purified by flash chromatography (1–3 % MeOH in CHCl_3_), and the red fractions were collected. These were concentrated, redissolved in CHCl_3_ and extracted with water to yield the product as a red solid (77 mg, 0.034 mmol, 25 %); ^1^H NMR (300 MHz, acetone): *δ*=2.10 (app. s, 108 H), 2.81–3.07 (m, 24 H), 4.79–5.07 (m, 24 H), 7.89 (dd, *J=*15.8, 6.6 Hz, 12 H), 8.37 (dd, *J=*15.8, 8.5 Hz, 12 H), 8.82–8.98 ppm (m, 12 H); ^13^C NMR (126 MHz, CDCl_3_): *δ*=27.9, 28.1, 29.6, 37.1, 50.9, 81.4, 82.4, 123.5, 143.5, 157.2, 157.3, 162.2, 169.2, 169.4, 170.0 ppm; UV/Vis (MeOH): *λ*_max_ (*ϵ*)=306 nm (240, 723, 981 mol^−1^ dm^3^ cm^−1^); HRMS (ESI): *m*/*z* calcd for [C_108_H_150_N_12_O_30_Ru]^2+^: 1098.4822; found: 1098.4854

**Tris ((2*S*,2′*R*)‐2,2′‐(((2,2′‐bipyridine)‐4,4′‐dicarbonyl)bis(azanediyl))disuccinic acid)ruthenium(II) ditrifluoroacetate (complex 1)**: Tris ((2*S*,2′*R*)‐tetra‐*tert*‐butyl‐2,2′‐(((2,2′‐bipyridine)‐4,4′‐dicarbonyl)bis(azanediyl))disuccinate)ruthenium(II) dinitrate (68 mg), TFA (4.5 mL) and water (0.5 mL) were stirred for three days. The reaction mixture was then concentrated in vacuo to yield the product as a red‐black solid (57 mg, 0.0294 mmol, 98 %); ^1^H NMR (500 MHz, D_2_O): *δ*=8.97 (s, 1 H), 7.90 (s, 1 H), 7.70 (s, 1 H), 4.61 (s, 1 H), 2.78 (s, 1 H), 2.67 ppm (s, 1 H); IR (solid state): $\tilde{\nu }$ =3182, 3050, 1648 cm^−1^; HRMS (ESI): *m*/*z* calcd for [C_60_H_54_N_12_O_30_Ru]^2+^: 762.1056; found: 762.1081.

**Protein expression and purification**: Cytochrome *c* peroxidase (CCP) was overexpressed in *Escherichia coli* by using the plasmid pT7CCP), in which expression was placed under the control of T7 RNA polymerase. The enzyme was isolated from *E. coli* BL21(DE3) as an apo‐enzyme, which was purified according to the literature.^1^ A 2 L culture of the expression strain supplemented with ampicillin was grown at 37 °C for 36 h in a medium containing (per litre) bactotryptone (10 g), yeast extract (8 g), NaCl (5 g), glycerol (1 mL), and ampicillin (100 mg). Subsequent steps were performed at 4 °C. The cells were harvested by centrifugation at 6000 *g* for 10 min, resuspended in buffer (40 mL) containing potassium phosphate (pH 7.5, 200 mm), Roche protease inhibitor tablets mini (2 tablets) and EDTA (1 mm), and lysed by passing through a cell disrupter. The lysate was diluted with cold H_2_O (100 mL). Enough ascorbic acid was added to bring the buffer to 5 mm. To improve the ratio of the Soret band to the band at 280 nm, an excess of haem was added: 80 mg of haemin/12 L culture was dissolved in a minimal amount of KOH (100 mm) in the dark, and diluted tenfold with potassium phosphate (pH 6, 100 mm). The haem solution was gradually added to clarified lysate on ice over 30 min with gentle stirring, then stirred on ice for 1 h in the dark. The excess haem was then precipitated by firstly acidifying the solution with acetic acid (100 mm) to pH 5.0 and then freezing the solution in dry ice until just frozen. The solution was then allowed to thaw with gentle shaking at 37 °C, then centrifuged at 12 000 *g* for 20 min, and the supernatant was decanted. The clear supernatant was loaded onto a DEAE‐Sepharose CL‐6B (3×5 cm) column equilibrated with potassium phosphate (pH 6, 50 mm) and washed with the same buffer. After elution with potassium phosphate (pH 6, 500 mm) the enzyme‐containing fractions were diluted with an equal volume of cold H_2_O and concentrated to approximately 1 mL by ultrafiltration (Amicon YM‐10 membrane). The sample was centrifuged at 12 000 *g* for 2 min to remove insoluble material, loaded onto a Sephadex G‐75 superfine column (3×60 cm) and eluted with potassium phosphate (pH 6, 100 mm) and EDTA (1 mm). The fractions containing A408/A280>1.1 were pooled (protein concentration was determined from the molar absorptivity; *ϵ*=55 mm ^−1^ cm^−1^, at 282 nm).

To exchange the haem for Zn porphyrin, the haem was removed by using the acid butanone method^2^ with minor modifications. Haem CCP solution (≈1 mm) in potassium phosphate buffer (100 mm) was diluted with four volumes of ice‐cold water. The CCP solution was adjusted to 100 mm fluoride by addition of KF solution (1 m), breaking the haem–protein linkage and turning the solution green. The haem was removed by lowering the pH of the solution to pH 3.2–3.3 by dropwise addition of ice‐cold HCl (0.1 m), with gentle stirring. The haem was then extracted by addition of an equal volume of ice‐cold butanone, shaking for 30 s and centrifugation for 1 min at 1000 *g*. The brown layer was siphoned away, and the extraction was repeated until the aqueous layer became colourless. The resulting apoCCP solution was diluted with a half volume of cold water and dialysed against two or three changes of NaHCO_3_ solution (10 mm). It was then dialysed against water, with the outer solution being changed every 2 h until there was no more discernible butanone (≈24 h), followed by dialysis into Tris**⋅**HCl (pH 7, 10 mm). A 4:1 excess of porphyrin was dissolved in KOH (100 mm, 200–500 μL) and diluted five to ten times with water. The porphyrin solution was added to the protein solution, and the protein solution was titrated to pH 7.8 with KOH (100 mm). In the dark, the alkaline porphyrin solution was added dropwise with gentle stirring to apoCCP until an approximately twofold excess of porphyrin was present. The solution was allowed to stand at near pH 8 for 20–30 min and then brought to pH 6.5–7.0 by addition of monobasic potassium phosphate (1 m). The protein was exchange into potassium phosphate (pH 6.5, 25 mm) and concentrated by ultracentrifugation to 0.5–1.0 mm CPP. The protein was loaded onto a small column of DEAE Sepharose CL‐6B, pre‐equilibrated with potassium phosphate (pH 6.5, 25 mm). The column was rinsed with around half a volume of loading buffer, and the metalloporphyrin CCP was eluted with potassium phosphate (pH 6.5, 0.6 m).

**Inhibition of Cyt** ***c*** **/CCP interaction determined by fluorescence recovery**: Cytochrome *c* (1 equiv), in a solution containing ZnCCP (2 μm), was added to a 500 μL micro fluorescence cell (Hellma Analytics) containing ZnCCP (2 μm, 500 μL, *ϵ*_280_=55 mm ^−1^ cm^−1^). Complex **2** (2 equiv) was then added. Fluorescence spectra were taken at each point (*λ*_ex_=430 nm). Separate comparative spectra for complex **2** were taken with use of identical instrument settings.

**Luminescence quenching assays**: All stocks for luminescence intensity assays were made up in phosphate buffer (pH 7.5, 5 mm). Ruthenium complex stocks were made up to 2 mm. Horse heart and yeast cyt *c* was obtained from Sigma–Aldrich and used without further purification. A cytochrome *c* stock was made up to ≈1 mm, and the concentration was accurately determined by using the molar extinction coefficients at 550 nm of 2.95×10^4^ mol^−1^ dm^3^ cm^−1^ for horse heart cyt *c* 56 and 2.11×10^4^ mol^−1^ dm^3^ cm^−1^ for yeast cyt *c* 56 after reduction by addition of one microspatula of sodium dithionite. Assays with oxidized cyt *c* in ascorbate‐containing buffer used cyt *c* oxidized with K_3_Fe(CN)_6_ followed by dialysis into buffer (pH 7.5, sodium phosphate (5 mm), sodium ascorbate (2 mm)) to remove the excess K_3_(CN)_6_. The concentration of oxidized cyt *c* was determined by using the molar extinction coefficient at 410 nm of 1.061×10^5^ mol^−1^ dm^3^ cm^−1^.^57^ All buffers used were at 5 mm concentration, pH 7.5, BSA (0.2 mg mL^−1^) unless otherwise stated.

Fluorimeter luminescence quenching assays were measured with a Jobin–Yvon Spex Fluorolog‐3 fluorimeter. Measurements were taken in a 4 mL quartz cuvette with excitation at 467 nm, and emission was measured over the 575–675 nm range, with 10 nm slit widths on both excitation and emission. Peak maxima were recorded over the entire cyt *c* concentration gradient.

Plate reader luminescence quenching assays were performed by using a PerkinElmer EnVision 2103 MultiLabel plate reader, with excitation at 467 nm, and emission at 630 nm fixed wavelength. A 2/3 dilution regime in a 384‐well plate (Optiplate) was used (total well volume 50 μL), with each result measured in triplicate. The *K*_d_ range possible for this assay is ≈5 nm–≈100 μm.

In all assays the ruthenium complex concentration was kept constant, with the concentration of cyt *c* being varied through the assay, as described below. Results obtained were fitted, by use of Origin9, to a 1:1 binding isotherm [Eq. (4)]:

(4) $$I=\frac{m[\left(a+b+K\right)-\sqrt{\left(a+b+K\right){}^{2}-4ab}]}{2a}$$

Where *I*=change in relative luminescence intensity (*I*/*I*_0_), *m*=maximum value of *I*, *a*=concentration of complex, *K*=dissociation constant, and *b*=concentration of protein added.

**Protein NMR**: Sensitivity‐enhanced ^1^H,^15^N HSQC NMR correlation spectra of ligand‐bound and unbound forms of cyt *c*, purchased from Sigma–Aldrich, were carried out at natural abundance with a 950 MHz Bruker Ascend Aeon spectrometer operating at a proton (^1^H) resonance frequency of 950.13 MHz and equipped with a Bruker TCI triple‐resonance cryoprobe. NMR acquisitions were carried out in buffer (pH 7.25, sodium phosphate (5 mm), sodium ascorbate (2 mm)). For cyt *c* alone, spectra were taken at 2 mm protein concentration. With complex **1**, cyt *c* (1 mm) and complex **1** (0.5 mm) were used, to a total volume of 600 μL. Spectra were analysed with the aid of the CcpNmr Analysis software package, and the chemical shift perturbations were calculated as the square roots of the sums of the isotope‐weighted shift differences squared [Eq. (5)]

(5) $$\Delta \delta =\sqrt{\left(\Delta \delta {}_{N}\right){}^{2}+\left(\gamma {}_{H}/\gamma {}_{N}\right){}^{2}\left(\Delta \delta {}_{H}\right){}^{2}}$$

where Δ*δ* is the overall change in chemical shift, Δ*δ*_N_ is the change in the nitrogen dimension, and Δ*δ*_H_ is the change in the proton dimension. The change in the proton dimension is scaled by the ratio of the gyromagnetic ratios of ^15^N (*γ*_N_) and ^1^H (*γ*_H_) to account for the larger chemical shift range of nitrogen.

## Conflict of interest

*The authors declare no conflict of interest*.

## Supporting information

As a service to our authors and readers, this journal provides supporting information supplied by the authors. Such materials are peer reviewed and may be re‐organized for online delivery, but are not copy‐edited or typeset. Technical support issues arising from supporting information (other than missing files) should be addressed to the authors.

Supplementary
