# Supplementary material for: Protein Surface Mimetics: Understanding How Ruthenium Tris(Bipyridines) Interact with Proteins
Source: Chembiochem. 2016 Dec 19;18(2):223–31. doi: 10.1002/cbic.201600552 (PMC5347857; doi:10.1002/cbic.201600552)
Supplement: Supplementary file 1 — Supplementary [file CBIC-18-223-s001.pdf]

CHEMBIOCHEM

Supporting Information

Protein Surface Mimetics: Understanding How Ruthenium Tris(Bipyridines) Interact with Proteins

Sarah H. Hewitt,[a, b] Maria H. Filby,[a, b] Ed Hayes,[a, b] Lars T. Kuhn,[b] Arnout P. Kalverda,[b] Michael E. Webb,[a, b] and Andrew J. Wilson*[a, b]

cbic_201600552_sm_miscellaneous_information.pdf

Table of Contents

1 Synthesis 2

1.1 General methods 2

1.2 Ru^{II} $(bpy)_3$ Synthesis 3

2 PPI inhibition 8

3 Assay data 9

1 Synthesis

1.1 General methods

Non-aqueous reactions were carried out in washed and oven-dried glassware. Solvents and reagents were used as supplied from major suppliers without prior purification unless stated. Anhydrous chloroform and dichloromethane were obtained from the in-house solvent purification system from Innovative Technology Inc. PureSolv®. Solvents used for reactions that are not anhydrous were of HPLC quality and provided by VWR, Fisher or Sigma-Aldrich. Water in aqueous solutions and quenching was deionised, Mixtures of solvents are quoted as ratios and correspond to a volume: volume ratio.

Thin layer (silica) chromatography was performed on Merck Kiesegel 60 F_{254} 0.25 mm precoated aluminium plates. Product spots were visualised under UV light (λ_{max} = 254 nm). Flash chromatography was performed using silica gel 60 (0.043 – 0.063 mm Sigma Aldrich) using pressure by means of head bellows.

^{1}H NMR spectra were recorded on Bruker DPX 300 (300 MHz) or Avance 500 (500 MHz) spectrometers and referenced to residual non-deuterated solvent peaks. ^{13}C spectra were recorded on a Bruker Avance 500 (126 MHz) and referenced to the solvent peak. ^{1}H spectra are reported as follows: ^{1}H NMR (spectrometer frequency, solvent) δ ppm to 2 d.p. (multiplicity, *J* coupling constant in Hertz, number of protons, assignments). Chemical shifts are quoted in ppm with signal splitting recorded as singlet (s), doublet (d), triplet (t), quartet (q), multiplet (m), broad (br). Coupling constants, *J*, are measured to the nearest 0.1 Hz. Similarly, ^{13}C spectra are reported as follows: *δ* (spectrometer frequency, solvent): ppm to one decimal place (assignment). Assignments of spectra were assisted by the results of DEPT, COSY, HMQC and HMBC experiments.

Infrared spectra were recorded on a Perkin Elmer Fourier-Transfer spectrometer. Spectra were analysed neat and only structurally important absorptions are quoted. Absorption maxima (ν_{max}) are quoted in wavenumbers (cm^{-1}).

Nominal mass spectra and accurate (4 d.p.) mass spectra were recorded on a Bruker Daltonics micrOTOF Premier Mass Spectrometer, under positive ESI conditions unless otherwise stated.

1.2 Ru^{II} (bpy)$_3$ Synthesis

1,4-Dimethyl (2S)-2-[(3-{[(2S)-1,4-dimethoxy-1,4-dioxobutan-2-yl]carbamoyl}-5-nitrophenyl)formamido]butanedioate

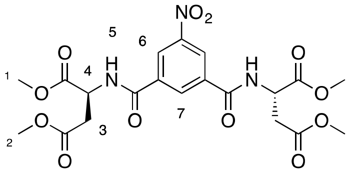

5-Nitroisophthalic acid (2.00 g, 9.47 mmol) and DMF (1 drop) in thionyl chloride (20 mL) was heated under reflux for 18 h. The solvent was evaporated to yield the acid chloride as a white solid. The acid chloride, dimethyl L-aspartic acid.HCl (4.12 g, 20.8 mmol) and triethylamine (2.92 mL, 20.8 mmol) in dry dichloromethane (40 mL) were stirred for 16 h. The resulting solution was quenched with sat. $NaHCO_3$ (50 mL) and the organic phase then washed with 1 M HCl (50 mL), and brine (50 mL). The organic phase was dried (Na_2SO_4) and concentrated to yield the crude product as a beige solid, this was purified by flash column chromatography (EtOAc/CH_2Cl_2 3:7) to yield the product as a white solid (2.54 g, 5.11 mmol, 54 %); ^1H NMR (500 MHz, $CDCl_3$) δ 3.03 (dd, *J* = 17.4, 6.1 Hz, 2 H, H3), 3.18 (dd, *J* = 17.4, 4.8 Hz, 2 H, H3'), 3.74 (s, 6 H, H1/H2), 3.84 (s, 6 H, H1/H2), 5.06 - 5.18 (m, 3 H, H4), 7.65 (d, *J* = 7.9 Hz, 3 H, H5), 8.60 (t, *J* = 1.5 Hz, 1 H, H7), 8.79 ppm (d, *J* = 1.5 Hz, 2 H, H6); IR (solid state, cm^{-1}) 3386 (N-H), 1747 (C=O); ESI-MS *m/z* found 498.1368 [*M*+H], [$C_{20}H_{24}N_3O_{12}$] requires 498.1354

1,4-Dimethyl (2S)-2-[(3-amino-5-{[(2S)-1,4-dimethoxy-1,4-dioxobutan-2-yl]carbamoyl}phenyl)formamido]butanedioate

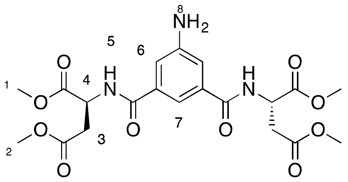

1,4-Dimethyl (2S)-2-[(3-{[(2S)-1,4-dimethoxy-1,4-dioxobutan-2-yl]carbamoyl}-5-nitrophenyl)formamido]butanedioate (1.00 g, 2.14 mmol) in methanol (30 mL) was degassed and palladium on activated charcoal (20 mg) was added, degassed again and put under a hydrogen atmosphere. The mixture was stirred for 18 h, then filtered twice and concentrated to yield the product as a cream solid (1.07 g, 2.29 mmol, quant.); ^{1}H NMR (500 MHz, $CDCl_3$) δ 3.00 (dd, J = 17.4, 4.9 Hz, 2 H, H3), 3.10 (dd, J = 17.4, 6.5 Hz, 2 H, H3'), 3.71 (s, 11 H, H1/H2), 3.80 (s, 11 H, H1/H2), 5.06 (dt, J = 6.5, 4.9 Hz, 4 H, H4), 7.18 (s, 2 H, H6), 7.45 (s, 1 H, H7), 7.46 - 7.48 ppm (m, 2 H, H8); ^{13}C NMR (126 MHz, $CDCl_3$) δ 36.1, 49.1, 52.2, 52.9, 115.0, 116.6, 135.1, 147.4, 166.7, 171.3, 171.6 ppm; IR (solid state, cm^{-1}) 3360, 1727, 1643, 1597; ESI-MS m/z found 468.1624 [M+H], [$C_{20}H_{26}N_3O_{10}$] requires 468.1613

1,4-Dimethyl (2*S*)-2-({3-[2-(4-{[3,5-bis({[(2*S*)-1,4-dimethoxy-1,4-dioxobutan-2-yl]carbamoyl})phenyl]carbamoyl}pyridin-2-yl)pyridine-4-amido]-5-{[(2*S*)-1,4-dimethoxy-1,4-dioxobutan-2-yl]carbamoyl}phenyl}formamido)butanedioate

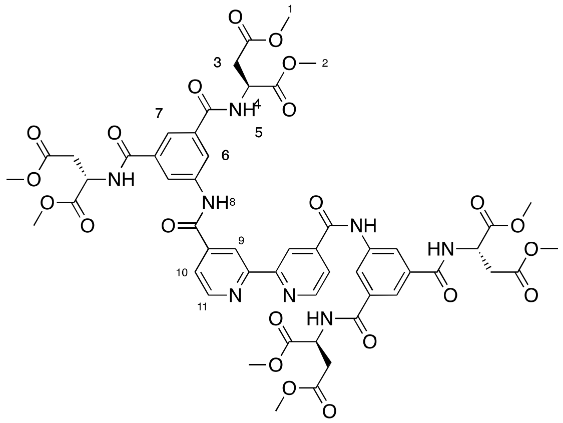

2,2'-Bipyridine 4,4'-dicarboxylic acid (471 mg, 1.93 mmol) in thionyl chloride (15 mL) was heated under reflux for 18 h. The solvent was then removed in vacuo, and the resulting acid chloride used immediately. The acid chloride was redissolved in anhydrous chloroform (10 mL) and heated to reflux, under N_2. A solution of 1,4-dimethyl (2S)-2-[(3-amino-5-{[(2S)-1,4-dimethoxy-1,4-dioxobutan-2-yl]carbamoyl} phenyl)formamido]butanedioate (1.62 g, 3.47 mmol) and anhydrous triethylamine (0.59 mL, 4.25 mmol) in anhydrous chloroform (20 mL), kept under N_2 was added dropwise to the refluxing solution and the resulting solution heated under reflux for 16 hrs. After which the reaction mixture was concentrated and the resulting pink crude product purified by flash column chromatography (5 % MeOH in chloroform) to yield the product as a beige solid (1.41 g, 1.23 mmol, 64 %); ^{1}H NMR (500 MHz, [D$_6$]DMSO) δ 2.90 (dd, *J* = 16.4, 8.0 Hz, 4 H, H3), 3.01 (dd, *J* = 16.4, 8.0 Hz, 4 H, H3'), 3.64 (s, 12 H, H1/H2), 3.67 (s, 12 H, H1/H2), 4.91 (app. q, *J* = 8.0 Hz, 4 H, H4), 8.06 (dd, *J* = 5.1, 1.3 Hz, 2 H, H10), 8.12 (s, 2 H, H7), 8.46 (d, *J*=1.3 Hz, 4 H, H6), 8.98 – 9.04 (m, 4 H, H9 and H11), 9.15 (d, *J*=7.6 Hz, 4 H, H5), 11.09 ppm (s, 2 H, H8); IR (solid state, cm^{-1}) 3306 (N-H), 3011 (N-H), 1731 (C=O), 1651 (C=O); ESI-MS *m/z* found 572.1751 [*M*+H]$^{2+}$, $[C_{52}H_{56}N_8O_{22}]^{2+}$ requires 572.1749

Tris (1,4-dimethyl (2S)-2-({3-[2-(4-{[3,5-bis({[(2S)-1,4-dimethoxy-1,4-dioxobutan-2-yl]carbamoyl})phenyl]carbamoyl}pyridin-2-yl)pyridine-4-amido]-5-{[(2S)-1,4-dimethoxy-1,4-dioxobutan-2-yl]carbamoyl}phenyl}formamido) butanedioate) ruthenium(II) dinitrate

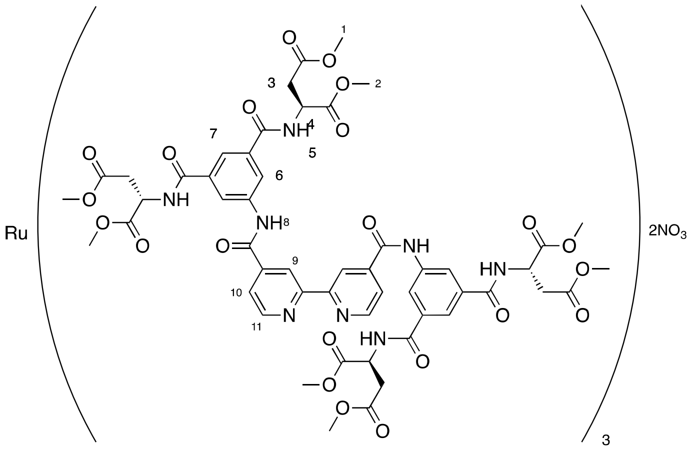

1,4-Dimethyl (2S)-2-({3-[2-(4-{[3,5-bis({[(2S)-1,4-dimethoxy-1,4-dioxobutan-2-yl]-carbamoyl})phenyl]carbamoyl}pyridin-2-yl)pyridine-4-amido]-5-{[(2S)-1,4-dimethoxy-1,4-dioxobutan-2-yl]carbamoyl}phenyl}formamido)butanedioate (1.00 g, 0.875 mmol), $Ru(DMSO)_4Cl_2$ (132 mg, 0.273 mmol) and silver nitrate (93 mg, 0.547 mmol) in ethanol (20 mL) were heated under reflux for 7 days. The reaction mixture was then filtered, and the red filtrate concentrated to yield the crude product as a red solid. This was purified by flash column chromatography (5 % - 10 % MeOH in CH_2Cl_2) to yield the product as a red solid (625 mg, 0.171 mmol, 63 %); ^1H NMR (500 MHz, $[D_6]DMSO$) δ 2.89 (dd, J = 16.3, 6.7 Hz, 12 H, H3), 2.99 (dd, J = 16.3, 6.7 Hz, 12 H, H3), 3.64 (s, 36 H, H1/H2), 3.67 (s, 36 H, H1/H2), 4.89 (q, J = 6.7 Hz, 7 H, H4), 8.06 - 8.16 (m, 12 H, H7 and H11), 8.18 (d, J = 5.9 Hz, 6 H, H10) 8.45 (s, 12 H, H6), 9.15 (d, J = 7.3 Hz, 6 H, H5), 9.58 (br. s., 6 H, H9), 11.23 ppm (s, 6 H, H8); IR (solid state, cm^{-1}) 3293 (N-H), 2953 (N-H), 1731 (C=O), 1656 (C=O)

Tris((2S)-2-({3-[2-(4-{[3,5-bis({[(1S)-1,2-dicarboxyethyl]carbamoyl})phenyl] carbamoyl}pyridin-2-yl)pyridine-4-amido]-5-{[(1S)-1,2-dicarboxyethyl] carbamoyl} phenyl}formamido)butanedioic acid) ruthenium dichloride, complex 2

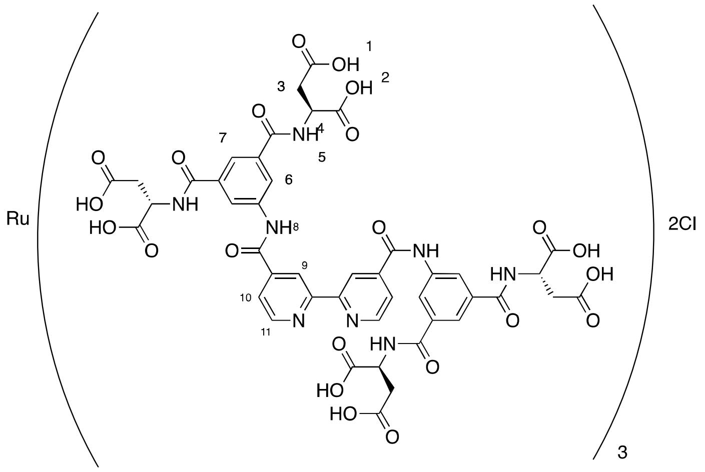

Tris(1,4-dimethyl (2S)-2-({3-[2-(4-{[3,5-bis({[(2S)-1,4-dimethoxy-1,4-dioxobutan-2-yl]carbamoyl})phenyl]carbamoyl}pyridin-2-yl)pyridine-4-amido]-5-{[(2S)-1,4-dimethoxy-1,4-dioxobutan-2-yl]carbamoyl}phenyl}formamido) butanedioate) ruthenium dinitrate (15 mg, 0.00411 mmol), lithium hydroxide (5 mg, 0.0205 mmol), THF (2 mL) and water (2 mL) were stirred for 1 h. The solution was then neutralized by addition of 1 M HCl. The red solution was concentrated, and was dialysed against pure water, to yield the product as a red solid (11 mg, 0.00328 mmol, 82 %); ^{1}H NMR (500 MHz, D_2O) δ 8.93 - 9.31 (br. s, 6 H, H9), 8.11 - 8.21 (br. s, 12 H, H6), 8.09 (br. d, *J*=5.4 Hz, 6 H, H11), 7.99 - 8.04 (br. s, 6 H, H7), 7.94 (d, *J*=5.4 Hz, 6 H, H10), 4.71 (m, 12 H, H4), 2.68 - 2.78 ppm (m, 24 H); ESI-HRMS found *m/z* 1596.211 [*M*]$^{2+}$, $C_{132}H_{114}N_{24}O_{66}Ru^{2+}$ requires 1596.2672

2 PPI inhibition

Agarose gels were prepared in 10 mM Tris-HCl buffer at pH 7.7 with 0.7% final agarose concentration. 40 µL wells were formed by placing a comb in the centre of the gel. After the proteins (10 µM, 20 µM for CCP in Lane 2 only) and the complex (20 µM) were mixed, 4µL of 80% glycerol was added to ensure proper well loading and a constant voltage of 100 V was applied for 20 min for sufficient separation. The gel was placed in staining solution (0.5% Coomassie blue, 40% methanol, 10% acetic acid aqueous solution) for 1h, followed by extensive destaining (40% methanol, 10% acetic acid aqueous solution) until protein bands were clear.

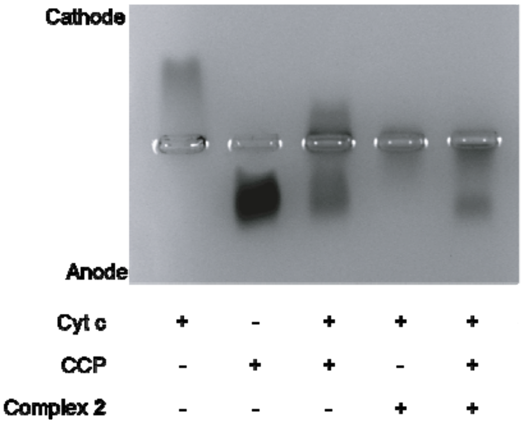

Figure S1. Native agarose gel (10 mM Tris-HCl buffer at pH 7.7 with 0.7% final agarose concentration), providing further evidence of PPI inhibition

Although this experiment is of low resolution, it provides supporting evidence for competitive PPI inhibition. Obtaining exact stoichiometric quantities of protein in agarose gels is challenging and hence dose response experiments were not attempted. Cyt *c* (positively charged) and CCP (negatively charged) migrate towards the cathode and anode respectively in isolation and this migration is retarded when both are present. The presence of the complex **2** retards the cathodic migration of cyt *c* but the anodic migration of CCP is less affected. The reason that CCP migrates towards the anode even

in the presence of cyt *c* may arise either because of mismatched stoichiometry between the two or because Cyt *c* does not fully "neutralize" charge on CCP. Whilst an alternative explanation for this experiment is that complex 2 binds to protein heterodimer without disruption the cyt c/ CCP interaction, in tandem with the luminescence data, it provides supporting evidence of competitive inhibition.

3 *Assay data*

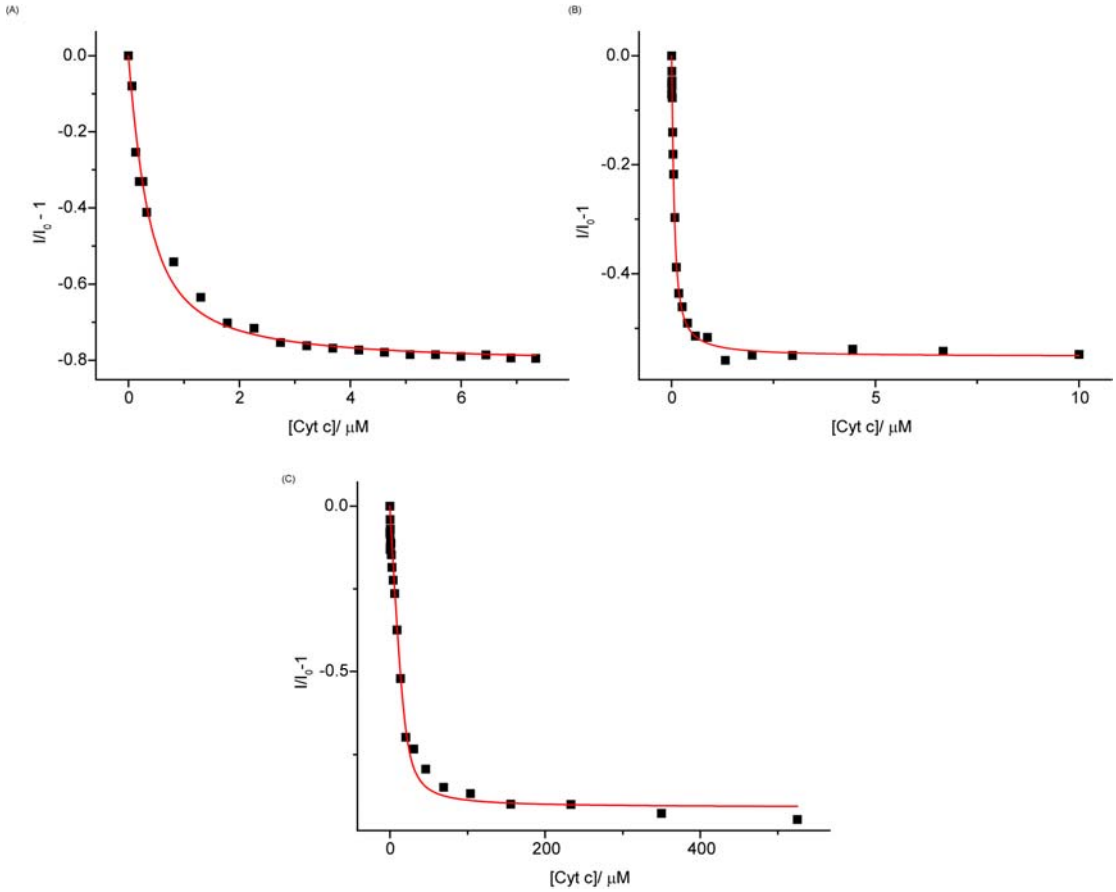

Figure S2. Exemplar binding curves are shown, for each complex (5 mM sodium phosphate, 0.2 mg mL^{-1} BSA, pH 7.5). (A) Binding curve for complex **1** on fluorometer (50 nM complex), (B) Binding curve for complex **1** on plate reader (50 nM complex), (C) Binding curve for complex **2** on plate reader (20 μM)

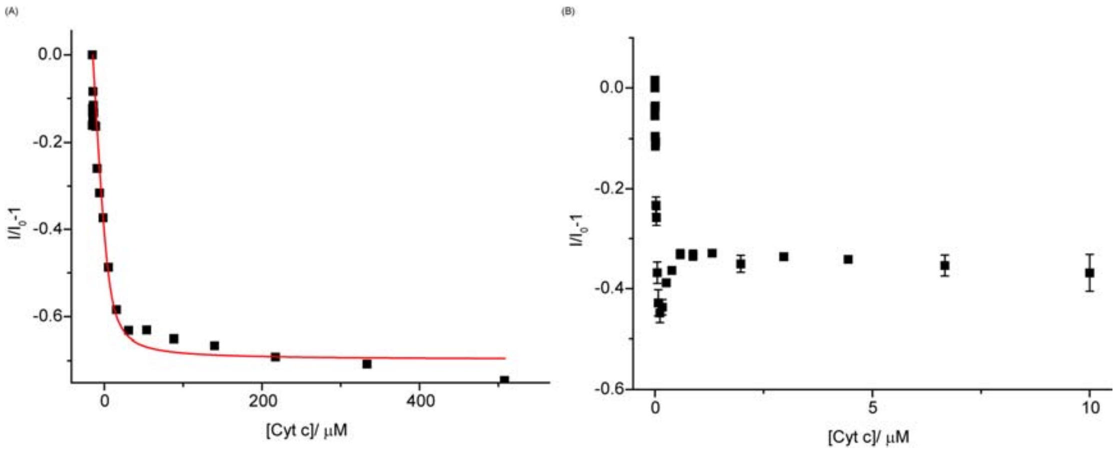

Figure S3. Binding isotherms for the complexes with yeast cyt *c* (5 mM sodium phosphate, 0.2 mg mL^{-1} BSA), (A) complex **1** (20 μM complex concentration, K_d = 2.81 ± 0.68 μM), (B) complex **2**, a different shape is observed, with an initial curve similar to that observed with horse heart cyt *c*, followed by an increase in luminescence
